# Crystal structure of limonoid TS3, isolated from *Trichilia rubescens*

**DOI:** 10.1107/S2056989018009775

**Published:** 2018-07-13

**Authors:** Patrice Kenfack Tsobnang, Armelle Tsamo Tontsa, Pierre Mkounga, Augustin Ephrem Nkengfack, Ignas Tonlé Kenfack

**Affiliations:** a Chemistry Department, University of Dschang, PO Box 67, Dschang, Cameroon; b Department of Organic Chemistry, University of Yaounde I, PO Box 812, Yaounde, Cameroon; c Department of Chemistry, Tshwane University of Technology, Pretoria 0001, South Africa

**Keywords:** crystal structure, limonoid, TS3, furan, Vilasinin derivatives, C—H⋯π inter­actions, hydrogen bonding

## Abstract

The title limonoid compound, C_26_H_28_O_5_·0.5H_2_O (**TS3**) [systematic name: (3a*S*,3b*S*,4a*S*,5a*S*,6*S*,7a*R*,8a*R*,8b*S*,11a*R*)-6-(furan-3-yl)-3a,5a,8b,11a-tetra­methyl-3a,4a,5,5a,6,7,7a,8b,11,11a-deca­hydro­oxireno[2′,3′:4b,5]oxireno[2′′,3′′:2′,3′]cyclo­penta­[1′,2′:7,8]phenanthro[10,1-*bc*]furan-3(3a*H*)-one hemihydrate], crystallizes with two independent mol­ecules (1 and 2) in the asymmetric unit and one water mol­ecule. **TS3** is composed of three six-membered rings (*A*, *C* and *D*), three five-membered rings (*B*, *E* and *F*) and two epoxide rings. A group of five fused rings (*A*–*E*) is bonded to a furan ring (*F*) with a C*sp* ^3^—C*sp* ^2^ bond [1.500 (3) Å in mol­ecule 1 and 1.499 (3) Å in mol­ecule 2]. The absolute structures of the mol­ecules in the crystal were determined by resonant scattering; Flack parameter = 0.05 (5). In the crystal, the individual mol­ecules stack in columns along the *b*-axis direction. The water mol­ecule bridges mol­ecules 1 and 2 *via* O_water_—H⋯O and C—H⋯O_water_ hydrogen bonds. Together with further C—H⋯O hydrogen bonds, linking mol­ecules 1 and 2, the columns are linked to form slabs parallel to the *ab* plane. Within each column, mol­ecules are also linked *via* C—H⋯π inter­actions involving the five-membered furan (*F*) rings.

## Abstract 2

The absolute configurations of the 10 asymmetric carbons involved in the structure of the title limonoid, **TS3**, have been confirmed by resonant scattering. The roles of the water mol­ecules and the C—H⋯π inter­actions in the crystal packing are highlighted.

## Chemical context   

Limonoids are a prominent class of secondary metabolites found in plants of the Meliaceae and Rutaceae families. They are also well known for their wide range of bioactive compounds that exhibit anti­plasmodial, anti­viral, anti­tumoral, anti­bacterial and cytotoxic properties (Krief *et al.*, 2004 ▸; Lange *et al.*, 2016 ▸). Vilasinin is one of the limonoid classes, to which belongs the title compound (TS3), and all the compounds of the rubescin series have been isolated from *Trichilia rubescens* (Tontsa *et al.*, 2013 ▸; Tsamo *et al.*, 2016 ▸). Among the broad spectrum of biological properties exhibited by vilasinin deriv­atives, **TS3** has been found to induce apoptosis in human hepatoma cell lines, to inter­fere with NFkB signaling and to enhance cAMP-regulated chloride conductance of cells expressing CFTR (cystic fibrosis transmembrane conductance regulator) (deCa­rvalho *et al.*, 2002 ▸).

As a result of the structure–activity relationships existing between bioactive compounds from the same series and/or class (Bauer *et al.*, 2001 ▸; Ariëns, 1986 ▸), it is important to fully characterize each bioactive mol­ecule. The mol­ecular structure of **TS3** was previously elucidated by one- and two-dimensional NMR techniques in combination with high-resolution mass spectrometry (deCa­rvalho *et al.*, 2002 ▸). However, the absolute configurations of the asymmetric carbons involved in its structure were not reported, and to date, no work on the crystal structure of this mol­ecule is known. Herein, we report the crystal structure of limonoid **TS3** and the roles of the water mol­ecules and the C—H⋯π inter­actions involving the furan rings in the crystal packing.
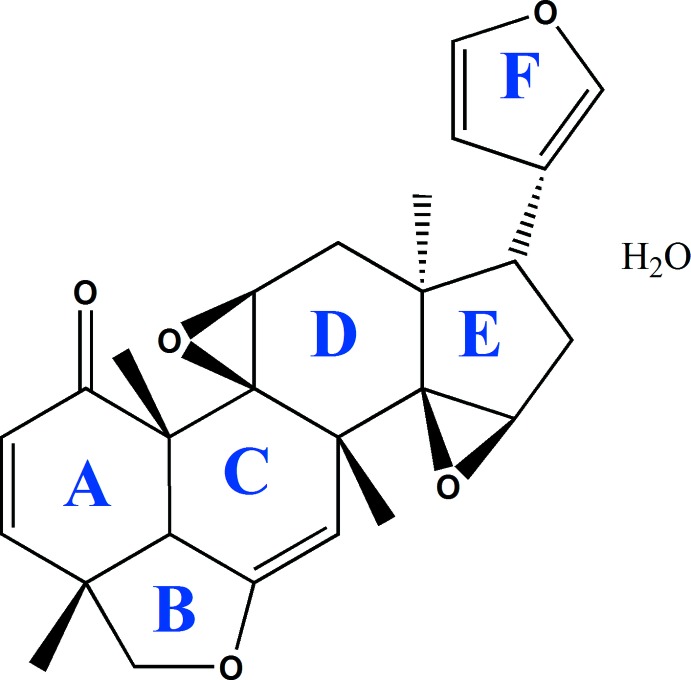


## Structural commentary   

**Figure 1:**
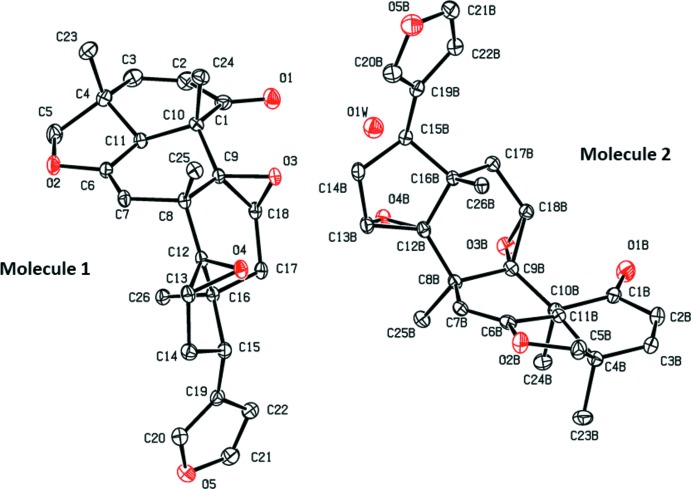
The mol­ecular structure of the two independent mol­ecules (1 and 2) of the title compound **TS3** with the crystallographic labelling scheme. Displacement ellipsoids are drawn at the 50% probability level. H atoms have been omitted for clarity.

**Figure 2:**
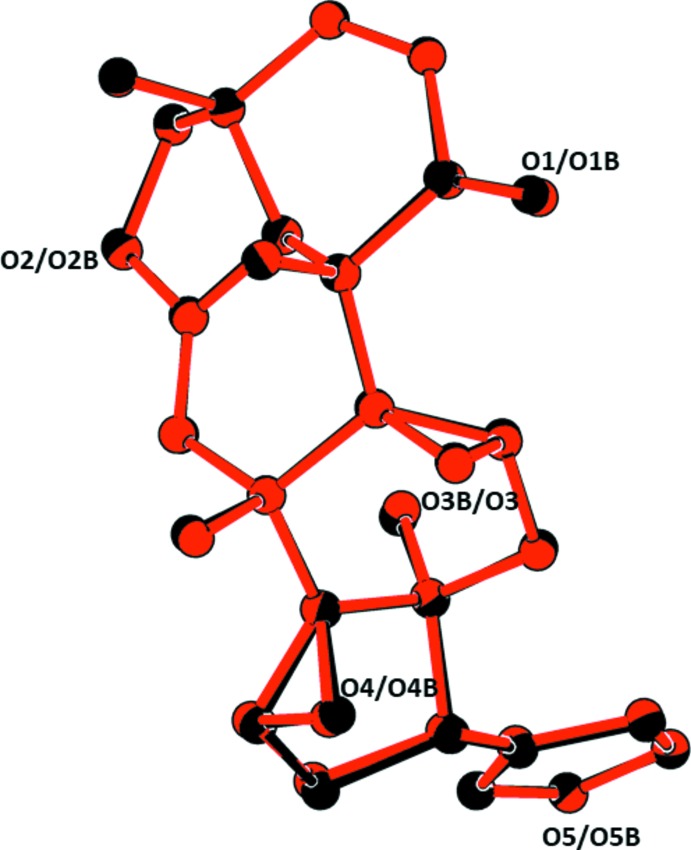
A view of the AutoMolFit (*PLATON*; Spek, 2009 ▸) of the two independent mol­ecules of **TS3** (colour code: black = mol­ecule 1, red = mol­ecule 2).

The asymmetric unit of the title compound contains one water mol­ecule and two crystallographically independent mol­ecules (1 and 2) of **TS3**, as illustrated in Fig. 1 ▸. The two mol­ecules are very similar with an r.m.s. fit of 0.068 Å for the 31 non-H atoms (Fig. 2 ▸).

As previously reported, using one- and two-dimensional NMR techniques in combination with high-resolution mass spectroscopy studies (deCa­rvalho *et al.*, 2002 ▸), the **TS3** mol­ecule consists of three six-membered rings (*A*, *C* and *D*), three five-membered rings (*B*, *E* and *F*), and two epoxide rings. Rings *A* to *E* are fused (first compartment), while ring *F* is bonded to this first moiety by a C*sp* ^3^—C*sp* ^2^ bond, [C15—C19 = 1.500 (3) Å and C15*B*—C19*B* = 1.499 (3) Å], as shown in Fig. 1 ▸.

The six-membered rings *A* and *D* have envelope conformations with atoms C11/C11*B* and C16/C16*B*, respectively, as the flaps, being displaced from the mean plane of the other five atoms by 0.657 (2)/0.672 (2) Å for atoms C11/C11*B* and by 0.654 (2)/0.670 (2) Å for atoms C16/C16*B*. The six-membered ring *C* has a half-chair conformation in both mol­ecules; the puckering parameters for mol­ecule 1 are amplitude *Q* = 0.474 (2) Å, θ = 131.7 (2)° and φ = 40.9 (3)°, while for mol­ecule 2 *Q* = 0.479 (2) Å, θ = 127.5 (2)°, φ = 42.9 (3)°. The five-membered rings *B* and *E* have envelope conformations with atoms C4/C4*B* and C15/C15*B*, respectively, as the flaps, being displaced from the mean plane of the other four atoms by 0.689 (2)/0.702 (2) Å and 0.526 (2)/0.454 (2) Å, respectively. The furan rings (*F*), are planar in both mol­ecules.

The chirality of **TS3** comes from ten asymmetric carbon atoms (C4, C8, C9, C10, C11, C12, C13, C15, C16 and C18; see Fig. 1 ▸), which have the following absolute configurations 4*R*, 8*S*, 9*S*, 10*S*, 11*S*, 12*R*, 13*R*, 15*S*, 16*S* and 18*S*. This has been confirmed by resonant scattering; Flack parameter = 0.05 (5), refined using Cu *K*α radiation.

## Supra­molecular features   

**Figure 3:**
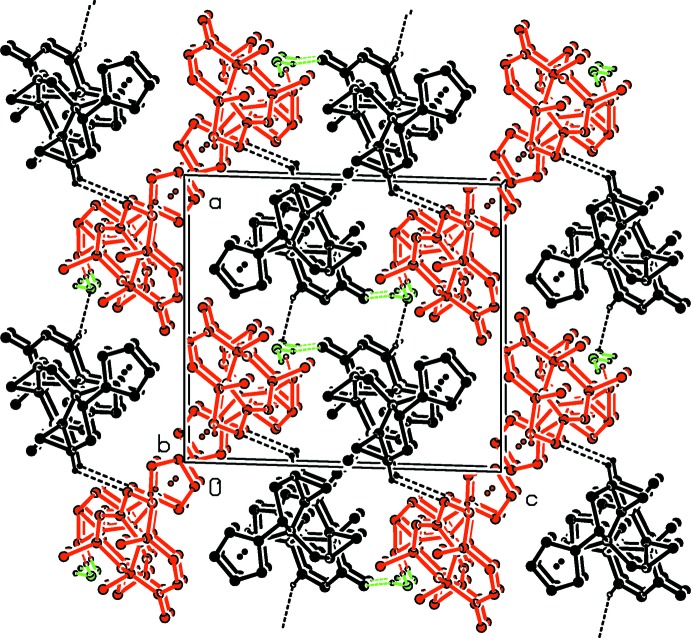
A view along the *b* axis of the crystal packing of the title compound. The hydrogen bonds are shown as dashed lines (see Table 1 ▸; colour code: black = mol­ecule 1, red = mol­ecule 2, green = water mol­ecule). For clarity, only the H atoms involved in hydrogen bonding have been included.

There are a number of hydrogen-bonding acceptor atoms (ketone and epoxide functions) present in the structure of **TS3**, and details are given in Table 1 ▸. The water mol­ecule of the asymmetric unit contributes significantly to the crystal packing *via* three weak hydrogen bonds (Fig. 3 ▸ and Table 1 ▸). The individual mol­ecules stack in columns along the *b*-axis direction, and within each column there are C—H⋯π_furan_ inter­actions present (Table 1 ▸), stabilizing the columnar structures. Mol­ecules 1 (black in Fig. 3 ▸) are linked about a twofold screw axis, *via* O_water_—H⋯O and C—H⋯O_water_ hydrogen bonds, forming helices propagating along the *b*-axis direction. Mol­ecules 1 and 2 (red in Fig. 3 ▸) are linked by O_water_—H⋯O hydrogen bonds (water is green in Fig. 3 ▸; see Table 1 ▸) and C—H⋯O hydrogen bonds, so forming slabs lying parallel to the *ab* plane. There are no other significant inter­molecular inter­actions present in the crystal structure.

## Database survey   

A search in the Cambridge Structural Database (CSD, Version 5.39, update May 2018; Groom *et al.*, 2016 ▸) for the skeleton of **TS3** gave no hits. The moieties having the rings *E* and *F* have been seen in three cytotoxic limonoids, *viz*. aphanastatine, amoorastatine and hydroxyl-12-ammorastatine (Arnoux & Pascard, 1980 ▸; Polonsky *et al.*, 1978 ▸). This moiety is also involved in the structure of Munronin H (Yan *et al.* 2015 ▸) and Toosendanin (Xu & Zhang, 2011 ▸). A number of structures with the second moiety (the fused rings *A*, *B* and *C*), but having different substituents, are known. Most of these compounds are reported as hemisynthesis products, while **TS3** was obtained from a natural source.

## Extraction and crystallization   

The title compound was isolated from the root bark of *Trichilia rubescens*. The extraction and the isolation procedures were carried out according to the experimental protocols previously described by Tsamo *et al.* (2016 ▸). A small amount of **TS3** powder was dissolved in a mixture of *n*-hexa­ne–EtOAc (4:1) and needle-like crystals, suitable for single crystal X-ray diffraction analysis, were obtained by slow evaporation of the solvents at room temperature after three days.

## Refinement   

Crystal data, data collection and structure refinement details are summarized in Table 2 ▸. All hydrogen atoms could be located in difference-Fourier maps. During refinement, they were included in calculated positions and treated as riding: C—H = 0.93–0.98 Å with *U*_iso_(H) = 1.5*U*_eq_(C-meth­yl) and 1.2*U*_eq_(C) for other H atoms.

## Supplementary Material

Crystal structure: contains datablock(s) I, Global. DOI: 10.1107/S2056989018009775/su5448sup1.cif

Structure factors: contains datablock(s) I. DOI: 10.1107/S2056989018009775/su5448Isup2.hkl

CCDC reference: 1854616

Additional supporting information:  crystallographic information; 3D view; checkCIF report

## Figures and Tables

**Table 1:** Hydrogen-bond geometry (Å, °) *Cg*1 and *Cg*2 are the centroids of the furan rings O5/C19–C22 and O5*B*/C19*B*–C22*B*, respectively.

*D*—H⋯*A*	*D*—H	H⋯*A*	*D*⋯*A*	*D*—H⋯*A*
O1*W*—H1*W*⋯O4*B*	0.91 (4)	1.95 (4)	2.857 (2)	171 (4)
O1*W*—H2*W*⋯O1	0.92 (4)	1.93 (4)	2.838 (2)	166 (3)
C15*B*—H15*B*⋯O1*W*	0.98	2.47	3.408 (3)	160
C3—H3⋯O1*W* ^i^	0.93	2.32	3.155 (3)	149
C13—H13⋯O2*B* ^ii^	0.98	2.47	3.088 (2)	121
C5—H5*A*⋯*Cg*1^iii^	0.97	2.93	3.744 (3)	142
C5*B*—H5*C*⋯*Cg*2^iv^	0.97	2.91	3.806 (3)	154

Symmetry codes: (i) 
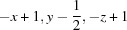
; (ii) 
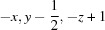
; (iii) 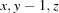; (iv) 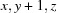.

**Table 2:** Experimental details

Crystal data	
Chemical formula	C_26_H_28_O_5_·0.5H_2_O
*M*_r_	429.49
Crystal system, space group	Monoclinic, *P*2_1_
Temperature (K)	293
*a*, *b*, *c* (Å)	12.4711 (2), 12.0986 (2), 13.7645 (2)
β (°)	91.742 (1)
*V* (Å^3^)	2075.87 (6)
*Z*	4
Radiation type	Cu *K*α
μ (mm^−1^)	0.78
Crystal size (mm)	0.24 × 0.17 × 0.11

Data collection	
Diffractometer	Bruker D8 Venture Photon
Absorption correction	Multi-scan (*SADABS*; Bruker, 2013 ▸)
*T*_min_, *T*_max_	0.869, 0.900
No. of measured, independent and observed [*I* > 2σ(*I*)] reflections	33504, 8304, 8067
*R*_int_	0.037
(sin θ/λ)_max_ (Å^−1^)	0.631

Refinement	
*R*[*F* ^2^ > 2σ(*F* ^2^)], *wR*(*F* ^2^), *S*	0.031, 0.080, 1.02
No. of reflections	8304
No. of parameters	585
No. of restraints	3
H-atom treatment	H atoms treated by a mixture of independent and constrained refinement
Δρ_max_, Δρ_min_ (e Å^−3^)	0.24, −0.24
Absolute structure	Flack *x* determined using 3527 quotients [(*I* ^+^)−(*I* ^−^)]/[(*I* ^+^)+(*I* ^−^)] (Parsons *et al.*, 2013 ▸)
Absolute structure parameter	−0.05 (5)

Computer programs: *APEX2* (Bruker, 2013 ▸), *SAINT* (Bruker, 2013 ▸), *SHELXS97* (Sheldrick, 2008 ▸), *PLATON* (Spek, 2009 ▸), *SHELXL2018* (Sheldrick, 2015 ▸), *publCIF* (Westrip, 2010 ▸) and *enCIFer* (Allen *et al.*, 2004 ▸).

## supplementary crystallographic information

### Crystal data

**Table:**

C_26_H_28_O_5_·0.5H_2_O	*F*(000) = 916
*M**_r_* = 429.49	*D*_x_ = 1.374 Mg m^−^^3^
Monoclinic, *P*2_1_	Cu *K*α radiation, λ = 1.54184 Å
*a* = 12.4711 (2) Å	Cell parameters from 30836 reflections
*b* = 12.0986 (2) Å	θ = 3.6–76.5°
*c* = 13.7645 (2) Å	µ = 0.78 mm^−^^1^
β = 91.742 (1)°	*T* = 293 K
*V* = 2075.87 (6) Å^3^	Needle, colourless
*Z* = 4	0.24 × 0.17 × 0.11 mm

### Data collection

**Table:**

Bruker D8 Venture Photon diffractometer	8304 independent reflections
Radiation source: fine-focus sealed tube	8067 reflections with *I* > 2σ(*I*)
Graphite monochromator	*R*_int_ = 0.037
ω scans	θ_max_ = 76.5°, θ_min_ = 3.6°
Absorption correction: multi-scan (SADABS; Bruker, 2013)	*h* = −15→15
*T*_min_ = 0.869, *T*_max_ = 0.900	*k* = −14→14
33504 measured reflections	*l* = −17→17

### Refinement

**Table:**

Refinement on *F*^2^	Hydrogen site location: mixed
Least-squares matrix: full	H atoms treated by a mixture of independent and constrained refinement
*R*[*F*^2^ > 2σ(*F*^2^)] = 0.031	*w* = 1/[σ^2^(*F*_o_^2^) + (0.0454*P*)^2^ + 0.3923*P*] where *P* = (*F*_o_^2^ + 2*F*_c_^2^)/3
*wR*(*F*^2^) = 0.080	(Δ/σ)_max_ < 0.001
*S* = 1.02	Δρ_max_ = 0.24 e Å^−^^3^
8304 reflections	Δρ_min_ = −0.24 e Å^−^^3^
585 parameters	Extinction correction: (SHELXL2018; Sheldrick, 2015), Fc^*^=kFc[1+0.001xFc^2^λ^3^/sin(2θ)]^-1/4^
3 restraints	Extinction coefficient: 0.0012 (2)
Primary atom site location: structure-invariant direct methods	Absolute structure: Flack *x* determined using 3527 quotients [(*I*^+^)-(*I*^-^)]/[(*I*^+^)+(*I*^-^)] (Parsons *et al.*, 2013)
Secondary atom site location: difference Fourier map	Absolute structure parameter: −0.05 (5)

### Special details

**Table:**

Geometry. All esds (except the esd in the dihedral angle between two l.s. planes) are estimated using the full covariance matrix. The cell esds are taken into account individually in the estimation of esds in distances, angles and torsion angles; correlations between esds in cell parameters are only used when they are defined by crystal symmetry. An approximate (isotropic) treatment of cell esds is used for estimating esds involving l.s. planes.

### Fractional atomic coordinates and isotropic or equivalent isotropic displacement parameters (Å^2^)

**Table:**

	*x*	*y*	*z*	*U*_iso_*/*U*_eq_	
O1	0.41746 (12)	0.14419 (15)	0.43841 (11)	0.0241 (3)	
O2	0.16598 (12)	0.06854 (13)	0.78568 (10)	0.0198 (3)	
O3	0.25032 (11)	0.32523 (13)	0.45900 (9)	0.0153 (3)	
O4	0.07555 (11)	0.49060 (13)	0.55185 (9)	0.0156 (3)	
O5	0.32107 (13)	0.73009 (14)	0.90101 (10)	0.0230 (3)	
C1	0.37466 (15)	0.10520 (18)	0.50908 (14)	0.0165 (4)	
C2	0.42802 (16)	0.01223 (19)	0.56348 (16)	0.0212 (4)	
H2	0.496492	−0.007294	0.544749	0.025*	
C3	0.38682 (16)	−0.04584 (19)	0.63669 (15)	0.0208 (4)	
H3	0.425990	−0.103071	0.665632	0.025*	
C4	0.27696 (16)	−0.01778 (18)	0.67159 (14)	0.0177 (4)	
C5	0.25842 (17)	−0.0069 (2)	0.78109 (15)	0.0217 (4)	
H5A	0.242126	−0.078047	0.809516	0.026*	
H5B	0.320926	0.024129	0.814744	0.026*	
C6	0.16864 (15)	0.13711 (18)	0.70507 (13)	0.0150 (4)	
C7	0.10617 (14)	0.22341 (18)	0.68487 (13)	0.0148 (4)	
H7	0.051307	0.241959	0.726144	0.018*	
C8	0.12442 (14)	0.29238 (17)	0.59372 (13)	0.0131 (4)	
C9	0.23314 (14)	0.26303 (16)	0.54519 (13)	0.0125 (3)	
C10	0.26120 (14)	0.13921 (17)	0.54124 (13)	0.0136 (4)	
C11	0.26042 (15)	0.10364 (17)	0.64710 (12)	0.0140 (4)	
H11	0.322272	0.141257	0.677764	0.017*	
C12	0.12781 (14)	0.41522 (17)	0.62115 (12)	0.0131 (4)	
C13	0.03622 (15)	0.48258 (18)	0.65014 (13)	0.0153 (4)	
H13	−0.033175	0.447733	0.661699	0.018*	
C14	0.07544 (15)	0.57667 (18)	0.71357 (14)	0.0171 (4)	
H14A	0.067789	0.559555	0.781851	0.020*	
H14B	0.036630	0.644258	0.698299	0.020*	
C15	0.19491 (15)	0.58711 (17)	0.68820 (13)	0.0152 (4)	
H15	0.198080	0.629657	0.627769	0.018*	
C16	0.23002 (14)	0.46593 (17)	0.66484 (13)	0.0129 (3)	
C17	0.31596 (14)	0.45956 (17)	0.58806 (13)	0.0139 (4)	
H17A	0.300175	0.513536	0.537519	0.017*	
H17B	0.385212	0.477824	0.617818	0.017*	
C18	0.32133 (14)	0.34573 (17)	0.54313 (13)	0.0141 (4)	
H18	0.393621	0.314705	0.538894	0.017*	
C19	0.26341 (15)	0.64588 (18)	0.76324 (14)	0.0161 (4)	
C20	0.23689 (17)	0.67767 (19)	0.85381 (15)	0.0191 (4)	
H20	0.170304	0.665552	0.880527	0.023*	
C21	0.40322 (17)	0.73139 (19)	0.83680 (16)	0.0217 (4)	
H21	0.470499	0.762246	0.849459	0.026*	
C22	0.37255 (16)	0.68159 (18)	0.75275 (15)	0.0188 (4)	
H22	0.414055	0.672127	0.698369	0.023*	
C23	0.19303 (17)	−0.10012 (19)	0.62948 (16)	0.0227 (4)	
H23A	0.206321	−0.113130	0.562109	0.034*	
H23B	0.122426	−0.069795	0.635615	0.034*	
H23C	0.198207	−0.168588	0.664556	0.034*	
C24	0.18463 (16)	0.07846 (18)	0.46869 (14)	0.0180 (4)	
H24A	0.172018	0.123829	0.412280	0.027*	
H24B	0.117776	0.063956	0.499042	0.027*	
H24C	0.216565	0.009876	0.449725	0.027*	
C25	0.02811 (15)	0.27369 (18)	0.52141 (13)	0.0166 (4)	
H25A	0.044575	0.304156	0.459215	0.025*	
H25B	−0.034432	0.309465	0.545528	0.025*	
H25C	0.014639	0.195887	0.514783	0.025*	
C26	0.26491 (15)	0.40480 (18)	0.75841 (13)	0.0150 (4)	
H26A	0.286767	0.331023	0.742664	0.023*	
H26B	0.205918	0.401858	0.801544	0.023*	
H26C	0.323861	0.443330	0.789493	0.023*	
O1B	0.53795 (12)	0.83149 (15)	0.05648 (12)	0.0251 (3)	
O2B	0.10289 (11)	0.93782 (14)	0.17227 (11)	0.0208 (3)	
O3B	0.46495 (11)	0.66520 (13)	0.20786 (10)	0.0178 (3)	
O4B	0.29366 (11)	0.51414 (13)	0.33393 (10)	0.0169 (3)	
O5B	−0.00555 (14)	0.27653 (17)	0.02162 (12)	0.0305 (4)	
C1B	0.45539 (15)	0.87934 (18)	0.07558 (14)	0.0169 (4)	
C2B	0.41910 (17)	0.9757 (2)	0.01389 (15)	0.0211 (4)	
H2B	0.461209	0.993106	−0.038588	0.025*	
C3B	0.33216 (17)	1.03952 (18)	0.02651 (15)	0.0203 (4)	
H3B	0.316987	1.097712	−0.015861	0.024*	
C4B	0.25965 (16)	1.01621 (18)	0.10950 (14)	0.0175 (4)	
C5B	0.13681 (17)	1.00979 (19)	0.09317 (15)	0.0207 (4)	
H5C	0.104291	1.082378	0.097616	0.025*	
H5D	0.118139	0.977929	0.030238	0.025*	
C6B	0.18681 (14)	0.86786 (18)	0.19700 (13)	0.0151 (4)	
C7B	0.18892 (15)	0.78769 (17)	0.26239 (13)	0.0152 (4)	
H7B	0.130485	0.777578	0.301840	0.018*	
C8B	0.28611 (14)	0.71156 (17)	0.27387 (13)	0.0136 (4)	
C9B	0.37101 (14)	0.73245 (17)	0.19380 (13)	0.0132 (4)	
C10B	0.38751 (14)	0.85413 (17)	0.16550 (13)	0.0149 (4)	
C11B	0.27648 (15)	0.89395 (17)	0.13331 (13)	0.0137 (4)	
H11B	0.259989	0.854704	0.072388	0.016*	
C12B	0.24644 (15)	0.59214 (17)	0.26471 (13)	0.0136 (4)	
C13B	0.17889 (15)	0.53418 (18)	0.33388 (13)	0.0163 (4)	
H13B	0.147031	0.574508	0.387596	0.020*	
C14B	0.11593 (16)	0.44474 (19)	0.28065 (14)	0.0182 (4)	
H14C	0.044291	0.469985	0.262392	0.022*	
H14D	0.110654	0.378695	0.320162	0.022*	
C15B	0.18286 (15)	0.42311 (17)	0.19009 (13)	0.0154 (4)	
H15B	0.243299	0.375908	0.210517	0.018*	
C16B	0.22978 (14)	0.53904 (17)	0.16451 (13)	0.0133 (4)	
C17B	0.33879 (15)	0.53405 (18)	0.11715 (14)	0.0166 (4)	
H17C	0.382876	0.478287	0.149512	0.020*	
H17D	0.328889	0.512632	0.049558	0.020*	
C18B	0.39590 (14)	0.64461 (17)	0.12284 (13)	0.0145 (3)	
H18B	0.423868	0.671086	0.061268	0.017*	
C19B	0.12333 (16)	0.36456 (18)	0.10908 (14)	0.0177 (4)	
C20B	0.01969 (18)	0.3330 (2)	0.10555 (16)	0.0247 (4)	
H20B	−0.028675	0.347620	0.154017	0.030*	
C21B	0.0873 (2)	0.2725 (2)	−0.02906 (16)	0.0278 (5)	
H21B	0.093873	0.238839	−0.089331	0.033*	
C22B	0.16774 (18)	0.3238 (2)	0.02018 (15)	0.0223 (4)	
H22B	0.238141	0.331512	0.000811	0.027*	
C23B	0.2820 (2)	1.1007 (2)	0.19117 (16)	0.0248 (4)	
H23D	0.358042	1.109254	0.201233	0.037*	
H23E	0.251093	1.074955	0.250074	0.037*	
H23F	0.250762	1.170547	0.173178	0.037*	
C24B	0.44431 (16)	0.91718 (19)	0.24995 (15)	0.0199 (4)	
H24D	0.475454	0.983835	0.225597	0.030*	
H24E	0.499688	0.871524	0.278653	0.030*	
H4F	0.393081	0.935369	0.298107	0.030*	
C25B	0.33729 (16)	0.72721 (19)	0.37712 (13)	0.0189 (4)	
H25D	0.403559	0.686948	0.382147	0.028*	
H25E	0.289042	0.700066	0.424634	0.028*	
H25F	0.350935	0.804302	0.388451	0.028*	
C26B	0.14867 (15)	0.60459 (18)	0.10143 (13)	0.0159 (4)	
H26D	0.178448	0.675409	0.086058	0.024*	
H26E	0.083898	0.614713	0.136307	0.024*	
H26F	0.132897	0.564670	0.042432	0.024*	
O1W	0.40948 (13)	0.31544 (15)	0.29745 (12)	0.0260 (3)	
H1W	0.367 (3)	0.374 (3)	0.312 (3)	0.070 (14)*	
H2W	0.402 (3)	0.265 (3)	0.347 (3)	0.059 (12)*	

### Atomic displacement parameters (Å^2^)

**Table:**

	*U*^11^	*U*^22^	*U*^33^	*U*^12^	*U*^13^	*U*^23^
O1	0.0247 (7)	0.0238 (8)	0.0243 (7)	0.0063 (6)	0.0114 (6)	0.0030 (6)
O2	0.0255 (7)	0.0190 (8)	0.0152 (6)	0.0024 (6)	0.0045 (5)	0.0066 (5)
O3	0.0176 (6)	0.0175 (7)	0.0109 (6)	−0.0010 (5)	0.0030 (5)	0.0034 (5)
O4	0.0159 (6)	0.0182 (7)	0.0128 (6)	0.0043 (5)	0.0009 (5)	0.0051 (5)
O5	0.0288 (8)	0.0235 (9)	0.0166 (7)	−0.0010 (6)	0.0013 (6)	−0.0019 (6)
C1	0.0171 (9)	0.0144 (10)	0.0181 (8)	0.0010 (7)	0.0025 (7)	−0.0023 (7)
C2	0.0178 (9)	0.0193 (12)	0.0266 (10)	0.0044 (8)	0.0027 (7)	−0.0003 (8)
C3	0.0206 (9)	0.0165 (10)	0.0251 (10)	0.0047 (8)	−0.0013 (7)	0.0017 (8)
C4	0.0209 (9)	0.0147 (10)	0.0174 (9)	0.0005 (7)	0.0004 (7)	0.0028 (7)
C5	0.0262 (10)	0.0207 (11)	0.0183 (9)	0.0040 (8)	0.0010 (7)	0.0070 (8)
C6	0.0179 (8)	0.0153 (10)	0.0119 (8)	−0.0031 (7)	0.0009 (6)	0.0020 (7)
C7	0.0138 (8)	0.0182 (10)	0.0124 (8)	−0.0023 (7)	0.0028 (6)	0.0022 (7)
C8	0.0121 (7)	0.0157 (10)	0.0117 (8)	0.0001 (6)	0.0022 (6)	0.0018 (7)
C9	0.0137 (8)	0.0134 (10)	0.0105 (7)	0.0008 (7)	0.0019 (6)	0.0019 (6)
C10	0.0147 (8)	0.0144 (10)	0.0118 (8)	0.0006 (7)	0.0020 (6)	−0.0014 (7)
C11	0.0162 (8)	0.0142 (9)	0.0114 (8)	0.0003 (7)	−0.0006 (6)	0.0017 (7)
C12	0.0130 (8)	0.0159 (10)	0.0103 (7)	0.0021 (7)	0.0017 (6)	0.0030 (7)
C13	0.0138 (8)	0.0184 (10)	0.0139 (8)	0.0016 (7)	0.0032 (6)	0.0020 (7)
C14	0.0161 (9)	0.0173 (10)	0.0180 (8)	0.0027 (7)	0.0037 (7)	−0.0001 (7)
C15	0.0165 (9)	0.0147 (10)	0.0146 (8)	0.0013 (7)	0.0032 (6)	0.0022 (7)
C16	0.0126 (8)	0.0143 (10)	0.0119 (8)	0.0006 (6)	0.0020 (6)	0.0014 (6)
C17	0.0115 (7)	0.0164 (10)	0.0138 (8)	0.0001 (6)	0.0027 (6)	0.0031 (7)
C18	0.0123 (8)	0.0168 (10)	0.0135 (8)	0.0008 (7)	0.0032 (6)	0.0032 (7)
C19	0.0189 (9)	0.0129 (10)	0.0166 (8)	0.0018 (7)	0.0025 (7)	0.0034 (7)
C20	0.0211 (9)	0.0191 (10)	0.0174 (9)	−0.0006 (7)	0.0026 (7)	0.0010 (7)
C21	0.0224 (9)	0.0184 (11)	0.0244 (10)	−0.0016 (8)	0.0012 (8)	0.0004 (8)
C22	0.0206 (9)	0.0159 (10)	0.0201 (9)	−0.0010 (7)	0.0030 (7)	−0.0003 (7)
C23	0.0257 (10)	0.0157 (11)	0.0267 (10)	−0.0021 (8)	0.0013 (8)	0.0015 (8)
C24	0.0195 (9)	0.0188 (11)	0.0155 (8)	−0.0006 (7)	−0.0011 (7)	−0.0023 (7)
C25	0.0138 (8)	0.0201 (10)	0.0157 (8)	0.0001 (7)	−0.0006 (6)	−0.0001 (7)
C26	0.0168 (8)	0.0151 (10)	0.0133 (8)	−0.0007 (7)	0.0001 (6)	0.0013 (7)
O1B	0.0179 (7)	0.0265 (9)	0.0315 (8)	−0.0017 (6)	0.0100 (6)	0.0011 (7)
O2B	0.0187 (7)	0.0211 (8)	0.0230 (7)	0.0070 (6)	0.0066 (5)	0.0068 (6)
O3B	0.0137 (6)	0.0195 (8)	0.0200 (6)	0.0040 (5)	−0.0003 (5)	0.0007 (5)
O4B	0.0185 (6)	0.0177 (8)	0.0145 (6)	0.0006 (5)	−0.0009 (5)	0.0037 (5)
O5B	0.0307 (8)	0.0339 (10)	0.0265 (8)	−0.0114 (7)	−0.0049 (6)	−0.0029 (7)
C1B	0.0164 (8)	0.0162 (10)	0.0182 (9)	−0.0041 (7)	0.0043 (7)	−0.0012 (7)
C2B	0.0231 (9)	0.0209 (11)	0.0196 (9)	−0.0044 (8)	0.0063 (7)	0.0025 (8)
C3B	0.0293 (10)	0.0149 (10)	0.0169 (9)	−0.0028 (8)	0.0039 (7)	0.0024 (7)
C4B	0.0226 (9)	0.0150 (10)	0.0152 (8)	0.0016 (7)	0.0030 (7)	−0.0003 (7)
C5B	0.0235 (10)	0.0183 (11)	0.0203 (9)	0.0052 (8)	0.0033 (7)	0.0058 (8)
C6B	0.0139 (8)	0.0159 (10)	0.0157 (8)	0.0028 (7)	0.0021 (6)	−0.0030 (7)
C7B	0.0160 (8)	0.0153 (10)	0.0147 (8)	0.0016 (7)	0.0053 (6)	−0.0017 (7)
C8B	0.0147 (8)	0.0150 (10)	0.0113 (8)	0.0013 (7)	0.0023 (6)	0.0001 (7)
C9B	0.0103 (8)	0.0153 (10)	0.0141 (8)	0.0013 (7)	0.0008 (6)	−0.0002 (7)
C10B	0.0146 (8)	0.0153 (10)	0.0149 (8)	−0.0020 (7)	0.0027 (6)	−0.0007 (7)
C11B	0.0172 (8)	0.0127 (10)	0.0113 (7)	−0.0003 (7)	0.0024 (6)	−0.0001 (7)
C12B	0.0137 (8)	0.0160 (10)	0.0112 (7)	0.0027 (7)	0.0004 (6)	0.0022 (7)
C13B	0.0192 (9)	0.0176 (10)	0.0121 (8)	0.0004 (7)	0.0021 (6)	0.0029 (7)
C14B	0.0210 (9)	0.0200 (10)	0.0139 (8)	−0.0034 (7)	0.0033 (7)	0.0037 (7)
C15B	0.0181 (8)	0.0141 (10)	0.0139 (8)	−0.0014 (7)	0.0013 (7)	0.0019 (7)
C16B	0.0152 (8)	0.0133 (10)	0.0113 (8)	0.0002 (7)	0.0025 (6)	0.0011 (7)
C17B	0.0183 (9)	0.0147 (10)	0.0173 (8)	0.0009 (7)	0.0065 (7)	−0.0007 (7)
C18B	0.0135 (8)	0.0148 (10)	0.0153 (8)	0.0016 (7)	0.0037 (6)	0.0004 (7)
C19B	0.0236 (9)	0.0134 (10)	0.0161 (9)	−0.0011 (7)	0.0011 (7)	0.0031 (7)
C20B	0.0267 (10)	0.0255 (12)	0.0218 (10)	−0.0061 (9)	0.0000 (8)	−0.0016 (9)
C21B	0.0403 (12)	0.0265 (13)	0.0165 (9)	−0.0096 (10)	0.0009 (8)	−0.0019 (8)
C22B	0.0306 (10)	0.0198 (11)	0.0167 (9)	−0.0042 (8)	0.0028 (7)	0.0006 (8)
C23B	0.0379 (12)	0.0149 (11)	0.0218 (9)	0.0028 (8)	0.0011 (8)	−0.0037 (8)
C24B	0.0204 (9)	0.0196 (11)	0.0196 (9)	−0.0051 (7)	−0.0017 (7)	−0.0026 (8)
C25B	0.0241 (9)	0.0196 (11)	0.0128 (8)	−0.0010 (8)	−0.0009 (7)	−0.0004 (7)
C26B	0.0185 (8)	0.0162 (10)	0.0129 (8)	0.0005 (7)	−0.0003 (6)	0.0017 (7)
O1W	0.0249 (7)	0.0265 (9)	0.0269 (8)	0.0024 (6)	0.0039 (6)	0.0011 (7)

### Geometric parameters (Å, º)

**Table:**

O1—C1	1.219 (3)	O2B—C6B	1.380 (2)
O2—C6	1.387 (2)	O2B—C5B	1.466 (2)
O2—C5	1.473 (3)	O3B—C9B	1.435 (2)
O3—C9	1.427 (2)	O3B—C18B	1.453 (2)
O3—C18	1.457 (2)	O4B—C13B	1.452 (2)
O4—C13	1.456 (2)	O4B—C12B	1.453 (2)
O4—C12	1.459 (2)	O5B—C20B	1.370 (3)
O5—C20	1.373 (3)	O5B—C21B	1.371 (3)
O5—C21	1.373 (3)	C1B—C2B	1.504 (3)
C1—C2	1.496 (3)	C1B—C10B	1.551 (2)
C1—C10	1.551 (2)	C2B—C3B	1.346 (3)
C2—C3	1.343 (3)	C2B—H2B	0.9300
C2—H2	0.9300	C3B—C4B	1.505 (3)
C3—C4	1.504 (3)	C3B—H3B	0.9300
C3—H3	0.9300	C4B—C11B	1.528 (3)
C4—C11	1.520 (3)	C4B—C23B	1.538 (3)
C4—C5	1.537 (3)	C4B—C5B	1.544 (3)
C4—C23	1.545 (3)	C5B—H5C	0.9700
C5—H5A	0.9700	C5B—H5D	0.9700
C5—H5B	0.9700	C6B—C7B	1.323 (3)
C6—C7	1.327 (3)	C6B—C11B	1.476 (2)
C6—C11	1.472 (2)	C7B—C8B	1.527 (3)
C7—C8	1.530 (2)	C7B—H7B	0.9300
C7—H7	0.9300	C8B—C12B	1.531 (3)
C8—C12	1.534 (3)	C8B—C25B	1.552 (2)
C8—C25	1.553 (2)	C8B—C9B	1.572 (2)
C8—C9	1.570 (2)	C9B—C18B	1.483 (3)
C9—C18	1.488 (3)	C9B—C10B	1.538 (3)
C9—C10	1.540 (3)	C10B—C11B	1.519 (3)
C10—C11	1.520 (2)	C10B—C24B	1.544 (3)
C10—C24	1.546 (3)	C11B—H11B	0.9800
C11—H11	0.9800	C12B—C13B	1.469 (3)
C12—C13	1.468 (3)	C12B—C16B	1.530 (2)
C12—C16	1.522 (3)	C13B—C14B	1.513 (3)
C13—C14	1.507 (3)	C13B—H13B	0.9800
C13—H13	0.9800	C14B—C15B	1.543 (3)
C14—C15	1.546 (3)	C14B—H14C	0.9700
C14—H14A	0.9700	C14B—H14D	0.9700
C14—H14B	0.9700	C15B—C19B	1.499 (3)
C15—C19	1.500 (3)	C15B—C16B	1.564 (3)
C15—C16	1.566 (3)	C15B—H15B	0.9800
C15—H15	0.9800	C16B—C17B	1.527 (2)
C16—C17	1.530 (2)	C16B—C26B	1.534 (2)
C16—C26	1.537 (2)	C17B—C18B	1.516 (3)
C17—C18	1.512 (3)	C17B—H17C	0.9700
C17—H17A	0.9700	C17B—H17D	0.9700
C17—H17B	0.9700	C18B—H18B	0.9800
C18—H18	0.9800	C19B—C20B	1.347 (3)
C19—C20	1.355 (3)	C19B—C22B	1.445 (3)
C19—C22	1.439 (3)	C20B—H20B	0.9300
C20—H20	0.9300	C21B—C22B	1.345 (3)
C21—C22	1.349 (3)	C21B—H21B	0.9300
C21—H21	0.9300	C22B—H22B	0.9300
C22—H22	0.9300	C23B—H23D	0.9600
C23—H23A	0.9600	C23B—H23E	0.9600
C23—H23B	0.9600	C23B—H23F	0.9600
C23—H23C	0.9600	C24B—H24D	0.9600
C24—H24A	0.9600	C24B—H24E	0.9600
C24—H24B	0.9600	C24B—H4F	0.9600
C24—H24C	0.9600	C25B—H25D	0.9600
C25—H25A	0.9600	C25B—H25E	0.9600
C25—H25B	0.9600	C25B—H25F	0.9600
C25—H25C	0.9600	C26B—H26D	0.9600
C26—H26A	0.9600	C26B—H26E	0.9600
C26—H26B	0.9600	C26B—H26F	0.9600
C26—H26C	0.9600	O1W—H1W	0.91 (3)
O1B—C1B	1.217 (3)	O1W—H2W	0.92 (2)
			
C6—O2—C5	107.37 (15)	C9B—O3B—C18B	61.78 (12)
C9—O3—C18	62.10 (11)	C13B—O4B—C12B	60.74 (12)
C13—O4—C12	60.48 (11)	C20B—O5B—C21B	105.70 (18)
C20—O5—C21	105.99 (16)	O1B—C1B—C2B	119.27 (18)
O1—C1—C2	119.44 (18)	O1B—C1B—C10B	124.39 (19)
O1—C1—C10	123.41 (19)	C2B—C1B—C10B	116.16 (17)
C2—C1—C10	116.90 (17)	C3B—C2B—C1B	126.96 (19)
C3—C2—C1	126.46 (18)	C3B—C2B—H2B	116.5
C3—C2—H2	116.8	C1B—C2B—H2B	116.5
C1—C2—H2	116.8	C2B—C3B—C4B	119.69 (19)
C2—C3—C4	119.62 (19)	C2B—C3B—H3B	120.2
C2—C3—H3	120.2	C4B—C3B—H3B	120.2
C4—C3—H3	120.2	C3B—C4B—C11B	105.21 (16)
C3—C4—C11	105.43 (17)	C3B—C4B—C23B	109.30 (18)
C3—C4—C5	119.87 (17)	C11B—C4B—C23B	117.75 (17)
C11—C4—C5	96.37 (16)	C3B—C4B—C5B	121.03 (17)
C3—C4—C23	110.35 (18)	C11B—C4B—C5B	96.42 (16)
C11—C4—C23	117.01 (16)	C23B—C4B—C5B	107.26 (17)
C5—C4—C23	107.63 (17)	O2B—C5B—C4B	103.25 (15)
O2—C5—C4	103.68 (15)	O2B—C5B—H5C	111.1
O2—C5—H5A	111.0	C4B—C5B—H5C	111.1
C4—C5—H5A	111.0	O2B—C5B—H5D	111.1
O2—C5—H5B	111.0	C4B—C5B—H5D	111.1
C4—C5—H5B	111.0	H5C—C5B—H5D	109.1
H5A—C5—H5B	109.0	C7B—C6B—O2B	128.06 (17)
C7—C6—O2	127.60 (18)	C7B—C6B—C11B	124.13 (18)
C7—C6—C11	124.34 (17)	O2B—C6B—C11B	107.75 (17)
O2—C6—C11	107.88 (16)	C6B—C7B—C8B	120.80 (16)
C6—C7—C8	120.00 (17)	C6B—C7B—H7B	119.6
C6—C7—H7	120.0	C8B—C7B—H7B	119.6
C8—C7—H7	120.0	C7B—C8B—C12B	107.89 (15)
C7—C8—C12	109.30 (15)	C7B—C8B—C25B	108.93 (16)
C7—C8—C25	108.35 (15)	C12B—C8B—C25B	108.27 (16)
C12—C8—C25	108.29 (16)	C7B—C8B—C9B	112.33 (15)
C7—C8—C9	112.18 (15)	C12B—C8B—C9B	108.47 (15)
C12—C8—C9	107.83 (15)	C25B—C8B—C9B	110.83 (15)
C25—C8—C9	110.82 (15)	O3B—C9B—C18B	59.73 (12)
O3—C9—C18	59.97 (11)	O3B—C9B—C10B	117.51 (15)
O3—C9—C10	116.35 (15)	C18B—C9B—C10B	119.14 (15)
C18—C9—C10	119.00 (15)	O3B—C9B—C8B	112.27 (15)
O3—C9—C8	112.82 (15)	C18B—C9B—C8B	120.38 (17)
C18—C9—C8	120.29 (16)	C10B—C9B—C8B	115.44 (15)
C10—C9—C8	115.80 (15)	C11B—C10B—C9B	104.45 (15)
C11—C10—C9	103.50 (15)	C11B—C10B—C24B	117.13 (17)
C11—C10—C24	117.57 (16)	C9B—C10B—C24B	110.12 (16)
C9—C10—C24	110.41 (15)	C11B—C10B—C1B	102.69 (15)
C11—C10—C1	103.35 (15)	C9B—C10B—C1B	118.08 (16)
C9—C10—C1	118.59 (16)	C24B—C10B—C1B	104.75 (15)
C24—C10—C1	103.91 (15)	C6B—C11B—C10B	117.34 (16)
C6—C11—C10	118.01 (16)	C6B—C11B—C4B	103.46 (16)
C6—C11—C4	104.37 (16)	C10B—C11B—C4B	119.18 (16)
C10—C11—C4	118.79 (17)	C6B—C11B—H11B	105.2
C6—C11—H11	104.7	C10B—C11B—H11B	105.2
C10—C11—H11	104.7	C4B—C11B—H11B	105.2
C4—C11—H11	104.7	O4B—C12B—C13B	59.59 (12)
O4—C12—C13	59.67 (11)	O4B—C12B—C16B	111.08 (16)
O4—C12—C16	111.01 (16)	C13B—C12B—C16B	108.61 (16)
C13—C12—C16	108.54 (16)	O4B—C12B—C8B	115.81 (15)
O4—C12—C8	115.88 (15)	C13B—C12B—C8B	125.98 (17)
C13—C12—C8	126.08 (17)	C16B—C12B—C8B	120.35 (15)
C16—C12—C8	120.29 (16)	O4B—C13B—C12B	59.67 (11)
O4—C13—C12	59.85 (11)	O4B—C13B—C14B	112.21 (17)
O4—C13—C14	112.04 (16)	C12B—C13B—C14B	109.08 (15)
C12—C13—C14	109.59 (16)	O4B—C13B—H13B	120.3
O4—C13—H13	120.2	C12B—C13B—H13B	120.3
C12—C13—H13	120.2	C14B—C13B—H13B	120.3
C14—C13—H13	120.2	C13B—C14B—C15B	103.12 (15)
C13—C14—C15	103.23 (15)	C13B—C14B—H14C	111.1
C13—C14—H14A	111.1	C15B—C14B—H14C	111.1
C15—C14—H14A	111.1	C13B—C14B—H14D	111.1
C13—C14—H14B	111.1	C15B—C14B—H14D	111.1
C15—C14—H14B	111.1	H14C—C14B—H14D	109.1
H14A—C14—H14B	109.1	C19B—C15B—C14B	114.41 (16)
C19—C15—C14	114.58 (16)	C19B—C15B—C16B	115.89 (16)
C19—C15—C16	115.36 (16)	C14B—C15B—C16B	104.18 (16)
C14—C15—C16	104.32 (16)	C19B—C15B—H15B	107.3
C19—C15—H15	107.4	C14B—C15B—H15B	107.3
C14—C15—H15	107.4	C16B—C15B—H15B	107.3
C16—C15—H15	107.4	C17B—C16B—C12B	107.65 (15)
C12—C16—C17	107.62 (15)	C17B—C16B—C26B	111.05 (15)
C12—C16—C26	110.47 (15)	C12B—C16B—C26B	111.24 (16)
C17—C16—C26	111.61 (15)	C17B—C16B—C15B	113.88 (16)
C12—C16—C15	102.85 (15)	C12B—C16B—C15B	102.42 (14)
C17—C16—C15	113.27 (16)	C26B—C16B—C15B	110.26 (15)
C26—C16—C15	110.63 (15)	C18B—C17B—C16B	111.47 (16)
C18—C17—C16	111.66 (16)	C18B—C17B—H17C	109.3
C18—C17—H17A	109.3	C16B—C17B—H17C	109.3
C16—C17—H17A	109.3	C18B—C17B—H17D	109.3
C18—C17—H17B	109.3	C16B—C17B—H17D	109.3
C16—C17—H17B	109.3	H17C—C17B—H17D	108.0
H17A—C17—H17B	107.9	O3B—C18B—C9B	58.50 (11)
O3—C18—C9	57.93 (11)	O3B—C18B—C17B	117.30 (16)
O3—C18—C17	116.53 (15)	C9B—C18B—C17B	123.98 (16)
C9—C18—C17	124.25 (15)	O3B—C18B—H18B	115.0
O3—C18—H18	115.2	C9B—C18B—H18B	115.0
C9—C18—H18	115.2	C17B—C18B—H18B	115.0
C17—C18—H18	115.2	C20B—C19B—C22B	105.34 (19)
C20—C19—C22	105.37 (18)	C20B—C19B—C15B	127.87 (19)
C20—C19—C15	128.20 (18)	C22B—C19B—C15B	126.69 (18)
C22—C19—C15	126.43 (17)	C19B—C20B—O5B	111.6 (2)
C19—C20—O5	111.22 (18)	C19B—C20B—H20B	124.2
C19—C20—H20	124.4	O5B—C20B—H20B	124.2
O5—C20—H20	124.4	C22B—C21B—O5B	110.8 (2)
C22—C21—O5	110.43 (19)	C22B—C21B—H21B	124.6
C22—C21—H21	124.8	O5B—C21B—H21B	124.6
O5—C21—H21	124.8	C21B—C22B—C19B	106.6 (2)
C21—C22—C19	106.99 (18)	C21B—C22B—H22B	126.7
C21—C22—H22	126.5	C19B—C22B—H22B	126.7
C19—C22—H22	126.5	C4B—C23B—H23D	109.5
C4—C23—H23A	109.5	C4B—C23B—H23E	109.5
C4—C23—H23B	109.5	H23D—C23B—H23E	109.5
H23A—C23—H23B	109.5	C4B—C23B—H23F	109.5
C4—C23—H23C	109.5	H23D—C23B—H23F	109.5
H23A—C23—H23C	109.5	H23E—C23B—H23F	109.5
H23B—C23—H23C	109.5	C10B—C24B—H24D	109.5
C10—C24—H24A	109.5	C10B—C24B—H24E	109.5
C10—C24—H24B	109.5	H24D—C24B—H24E	109.5
H24A—C24—H24B	109.5	C10B—C24B—H4F	109.5
C10—C24—H24C	109.5	H24D—C24B—H4F	109.5
H24A—C24—H24C	109.5	H24E—C24B—H4F	109.5
H24B—C24—H24C	109.5	C8B—C25B—H25D	109.5
C8—C25—H25A	109.5	C8B—C25B—H25E	109.5
C8—C25—H25B	109.5	H25D—C25B—H25E	109.5
H25A—C25—H25B	109.5	C8B—C25B—H25F	109.5
C8—C25—H25C	109.5	H25D—C25B—H25F	109.5
H25A—C25—H25C	109.5	H25E—C25B—H25F	109.5
H25B—C25—H25C	109.5	C16B—C26B—H26D	109.5
C16—C26—H26A	109.5	C16B—C26B—H26E	109.5
C16—C26—H26B	109.5	H26D—C26B—H26E	109.5
H26A—C26—H26B	109.5	C16B—C26B—H26F	109.5
C16—C26—H26C	109.5	H26D—C26B—H26F	109.5
H26A—C26—H26C	109.5	H26E—C26B—H26F	109.5
H26B—C26—H26C	109.5	H1W—O1W—H2W	106 (4)
C6B—O2B—C5B	108.27 (15)		
			
O1—C1—C2—C3	−173.5 (2)	O1B—C1B—C2B—C3B	−177.9 (2)
C10—C1—C2—C3	1.0 (3)	C10B—C1B—C2B—C3B	−2.6 (3)
C1—C2—C3—C4	−0.8 (3)	C1B—C2B—C3B—C4B	−0.3 (3)
C2—C3—C4—C11	−26.3 (3)	C2B—C3B—C4B—C11B	−24.5 (3)
C2—C3—C4—C5	−133.3 (2)	C2B—C3B—C4B—C23B	102.9 (2)
C2—C3—C4—C23	100.9 (2)	C2B—C3B—C4B—C5B	−131.8 (2)
C6—O2—C5—C4	−28.9 (2)	C6B—O2B—C5B—C4B	−27.7 (2)
C3—C4—C5—O2	153.99 (19)	C3B—C4B—C5B—O2B	154.08 (19)
C11—C4—C5—O2	42.11 (19)	C11B—C4B—C5B—O2B	42.01 (18)
C23—C4—C5—O2	−78.9 (2)	C23B—C4B—C5B—O2B	−79.7 (2)
C5—O2—C6—C7	−173.6 (2)	C5B—O2B—C6B—C7B	−177.5 (2)
C5—O2—C6—C11	1.8 (2)	C5B—O2B—C6B—C11B	−0.2 (2)
O2—C6—C7—C8	177.46 (18)	O2B—C6B—C7B—C8B	174.55 (18)
C11—C6—C7—C8	2.8 (3)	C11B—C6B—C7B—C8B	−2.4 (3)
C6—C7—C8—C12	−130.80 (19)	C6B—C7B—C8B—C12B	−125.86 (19)
C6—C7—C8—C25	111.4 (2)	C6B—C7B—C8B—C25B	116.8 (2)
C6—C7—C8—C9	−11.3 (3)	C6B—C7B—C8B—C9B	−6.3 (3)
C18—O3—C9—C10	−109.79 (18)	C18B—O3B—C9B—C10B	−109.37 (18)
C18—O3—C9—C8	112.88 (17)	C18B—O3B—C9B—C8B	113.19 (18)
C7—C8—C9—O3	178.88 (15)	C7B—C8B—C9B—O3B	176.75 (15)
C12—C8—C9—O3	−60.73 (18)	C12B—C8B—C9B—O3B	−64.09 (19)
C25—C8—C9—O3	57.6 (2)	C25B—C8B—C9B—O3B	54.6 (2)
C7—C8—C9—C18	−113.64 (19)	C7B—C8B—C9B—C18B	−116.30 (19)
C12—C8—C9—C18	6.7 (2)	C12B—C8B—C9B—C18B	2.9 (2)
C25—C8—C9—C18	125.11 (18)	C25B—C8B—C9B—C18B	121.60 (19)
C7—C8—C9—C10	41.3 (2)	C7B—C8B—C9B—C10B	38.4 (2)
C12—C8—C9—C10	161.69 (14)	C12B—C8B—C9B—C10B	157.54 (15)
C25—C8—C9—C10	−80.0 (2)	C25B—C8B—C9B—C10B	−83.7 (2)
O3—C9—C10—C11	166.89 (15)	O3B—C9B—C10B—C11B	167.09 (15)
C18—C9—C10—C11	98.23 (18)	C18B—C9B—C10B—C11B	98.22 (17)
C8—C9—C10—C11	−57.04 (19)	C8B—C9B—C10B—C11B	−56.79 (18)
O3—C9—C10—C24	−66.45 (19)	O3B—C9B—C10B—C24B	−66.33 (19)
C18—C9—C10—C24	−135.10 (16)	C18B—C9B—C10B—C24B	−135.21 (17)
C8—C9—C10—C24	69.62 (19)	C8B—C9B—C10B—C24B	69.79 (19)
O3—C9—C10—C1	53.2 (2)	O3B—C9B—C10B—C1B	53.9 (2)
C18—C9—C10—C1	−15.4 (2)	C18B—C9B—C10B—C1B	−15.0 (2)
C8—C9—C10—C1	−170.71 (15)	C8B—C9B—C10B—C1B	−170.02 (15)
O1—C1—C10—C11	−160.1 (2)	O1B—C1B—C10B—C11B	−155.6 (2)
C2—C1—C10—C11	25.8 (2)	C2B—C1B—C10B—C11B	29.4 (2)
O1—C1—C10—C9	−46.3 (3)	O1B—C1B—C10B—C9B	−41.4 (3)
C2—C1—C10—C9	139.50 (18)	C2B—C1B—C10B—C9B	143.56 (18)
O1—C1—C10—C24	76.7 (2)	O1B—C1B—C10B—C24B	81.5 (2)
C2—C1—C10—C24	−97.54 (19)	C2B—C1B—C10B—C24B	−93.5 (2)
C7—C6—C11—C10	−23.6 (3)	C7B—C6B—C11B—C10B	−20.7 (3)
O2—C6—C11—C10	160.80 (17)	O2B—C6B—C11B—C10B	161.79 (16)
C7—C6—C11—C4	−158.03 (19)	C7B—C6B—C11B—C4B	−154.20 (19)
O2—C6—C11—C4	26.4 (2)	O2B—C6B—C11B—C4B	28.3 (2)
C9—C10—C11—C6	47.7 (2)	C9B—C10B—C11B—C6B	47.7 (2)
C24—C10—C11—C6	−74.3 (2)	C24B—C10B—C11B—C6B	−74.4 (2)
C1—C10—C11—C6	171.94 (17)	C1B—C10B—C11B—C6B	171.47 (17)
C9—C10—C11—C4	175.50 (16)	C9B—C10B—C11B—C4B	173.74 (15)
C24—C10—C11—C4	53.5 (2)	C24B—C10B—C11B—C4B	51.7 (2)
C1—C10—C11—C4	−60.2 (2)	C1B—C10B—C11B—C4B	−62.5 (2)
C3—C4—C11—C6	−164.54 (15)	C3B—C4B—C11B—C6B	−166.74 (15)
C5—C4—C11—C6	−41.12 (18)	C23B—C4B—C11B—C6B	71.2 (2)
C23—C4—C11—C6	72.4 (2)	C5B—C4B—C11B—C6B	−42.12 (17)
C3—C4—C11—C10	61.5 (2)	C3B—C4B—C11B—C10B	60.8 (2)
C5—C4—C11—C10	−175.07 (16)	C23B—C4B—C11B—C10B	−61.2 (2)
C23—C4—C11—C10	−61.5 (2)	C5B—C4B—C11B—C10B	−174.53 (15)
C13—O4—C12—C16	−99.79 (17)	C13B—O4B—C12B—C16B	−99.80 (17)
C13—O4—C12—C8	118.32 (18)	C13B—O4B—C12B—C8B	118.22 (19)
C7—C8—C12—O4	−140.84 (15)	C7B—C8B—C12B—O4B	−137.40 (15)
C25—C8—C12—O4	−23.0 (2)	C25B—C8B—C12B—O4B	−19.7 (2)
C9—C8—C12—O4	96.97 (17)	C9B—C8B—C12B—O4B	100.68 (17)
C7—C8—C12—C13	−70.8 (2)	C7B—C8B—C12B—C13B	−67.5 (2)
C25—C8—C12—C13	47.1 (2)	C25B—C8B—C12B—C13B	50.2 (2)
C9—C8—C12—C13	167.03 (16)	C9B—C8B—C12B—C13B	170.56 (17)
C7—C8—C12—C16	81.02 (19)	C7B—C8B—C12B—C16B	84.35 (19)
C25—C8—C12—C16	−161.14 (15)	C25B—C8B—C12B—C16B	−157.90 (16)
C9—C8—C12—C16	−41.2 (2)	C9B—C8B—C12B—C16B	−37.6 (2)
C12—O4—C13—C14	100.55 (18)	C12B—O4B—C13B—C14B	99.81 (17)
C16—C12—C13—O4	104.00 (16)	C16B—C12B—C13B—O4B	104.03 (16)
C8—C12—C13—O4	−101.50 (19)	C8B—C12B—C13B—O4B	−101.40 (19)
O4—C12—C13—C14	−104.71 (17)	O4B—C12B—C13B—C14B	−105.13 (18)
C16—C12—C13—C14	−0.7 (2)	C16B—C12B—C13B—C14B	−1.1 (2)
C8—C12—C13—C14	153.79 (17)	C8B—C12B—C13B—C14B	153.47 (17)
O4—C13—C14—C15	−43.4 (2)	O4B—C13B—C14B—C15B	−41.9 (2)
C12—C13—C14—C15	21.0 (2)	C12B—C13B—C14B—C15B	22.3 (2)
C13—C14—C15—C19	−159.66 (16)	C13B—C14B—C15B—C19B	−161.82 (17)
C13—C14—C15—C16	−32.58 (18)	C13B—C14B—C15B—C16B	−34.29 (19)
O4—C12—C16—C17	−75.67 (19)	O4B—C12B—C16B—C17B	−76.79 (19)
C13—C12—C16—C17	−139.46 (16)	C13B—C12B—C16B—C17B	−140.52 (17)
C8—C12—C16—C17	64.3 (2)	C8B—C12B—C16B—C17B	63.2 (2)
O4—C12—C16—C26	162.26 (15)	O4B—C12B—C16B—C26B	161.32 (15)
C13—C12—C16—C26	98.48 (18)	C13B—C12B—C16B—C26B	97.60 (19)
C8—C12—C16—C26	−57.8 (2)	C8B—C12B—C16B—C26B	−58.7 (2)
O4—C12—C16—C15	44.17 (18)	O4B—C12B—C16B—C15B	43.55 (18)
C13—C12—C16—C15	−19.61 (19)	C13B—C12B—C16B—C15B	−20.18 (19)
C8—C12—C16—C15	−175.85 (14)	C8B—C12B—C16B—C15B	−176.43 (15)
C19—C15—C16—C12	158.70 (15)	C19B—C15B—C16B—C17B	−84.0 (2)
C14—C15—C16—C12	32.11 (17)	C14B—C15B—C16B—C17B	149.37 (16)
C19—C15—C16—C17	−85.44 (19)	C19B—C15B—C16B—C12B	160.06 (16)
C14—C15—C16—C17	147.97 (15)	C14B—C15B—C16B—C12B	33.45 (18)
C19—C15—C16—C26	40.7 (2)	C19B—C15B—C16B—C26B	41.6 (2)
C14—C15—C16—C26	−85.86 (17)	C14B—C15B—C16B—C26B	−85.02 (17)
C12—C16—C17—C18	−47.9 (2)	C12B—C16B—C17B—C18B	−49.3 (2)
C26—C16—C17—C18	73.44 (19)	C26B—C16B—C17B—C18B	72.7 (2)
C15—C16—C17—C18	−160.92 (15)	C15B—C16B—C17B—C18B	−162.08 (15)
C9—O3—C18—C17	−115.44 (17)	C9B—O3B—C18B—C17B	−114.92 (18)
C10—C9—C18—O3	105.41 (17)	C10B—C9B—C18B—O3B	106.67 (17)
C8—C9—C18—O3	−100.44 (17)	C8B—C9B—C18B—O3B	−99.58 (17)
O3—C9—C18—C17	102.18 (19)	O3B—C9B—C18B—C17B	103.6 (2)
C10—C9—C18—C17	−152.41 (17)	C10B—C9B—C18B—C17B	−149.69 (18)
C8—C9—C18—C17	1.7 (3)	C8B—C9B—C18B—C17B	4.1 (3)
C16—C17—C18—O3	88.00 (18)	C16B—C17B—C18B—O3B	89.66 (19)
C16—C17—C18—C9	20.2 (2)	C16B—C17B—C18B—C9B	20.8 (3)
C14—C15—C19—C20	9.1 (3)	C14B—C15B—C19B—C20B	2.4 (3)
C16—C15—C19—C20	−112.1 (2)	C16B—C15B—C19B—C20B	−118.9 (2)
C14—C15—C19—C22	−170.2 (2)	C14B—C15B—C19B—C22B	−173.4 (2)
C16—C15—C19—C22	68.6 (3)	C16B—C15B—C19B—C22B	65.3 (3)
C22—C19—C20—O5	−0.3 (2)	C22B—C19B—C20B—O5B	−0.5 (3)
C15—C19—C20—O5	−179.66 (19)	C15B—C19B—C20B—O5B	−177.0 (2)
C21—O5—C20—C19	0.3 (2)	C21B—O5B—C20B—C19B	0.3 (3)
C20—O5—C21—C22	−0.2 (3)	C20B—O5B—C21B—C22B	0.0 (3)
O5—C21—C22—C19	0.1 (3)	O5B—C21B—C22B—C19B	−0.3 (3)
C20—C19—C22—C21	0.1 (2)	C20B—C19B—C22B—C21B	0.5 (3)
C15—C19—C22—C21	179.5 (2)	C15B—C19B—C22B—C21B	177.0 (2)

### Hydrogen-bond geometry (Å, º)

Cg1 and Cg2 are the centroids of the furan rings O5/C19–C22 and O5*B*/C19*B*–C22*B*, respectively.

**Table:**

*D*—H···*A*	*D*—H	H···*A*	*D*···*A*	*D*—H···*A*
O1*W*—H1*W*···O4*B*	0.91 (4)	1.95 (4)	2.857 (2)	171 (4)
O1*W*—H2*W*···O1	0.92 (4)	1.93 (4)	2.838 (2)	166 (3)
C15*B*—H15*B*···O1*W*	0.98	2.47	3.408 (3)	160
C3—H3···O1*W*^i^	0.93	2.32	3.155 (3)	149
C13—H13···O2*B*^ii^	0.98	2.47	3.088 (2)	121
C5—H5*A*···*Cg*1^iii^	0.97	2.93	3.744 (3)	142
C5*B*—H5*C*···*Cg*2^iv^	0.97	2.91	3.806 (3)	154

Symmetry codes: (i) −*x*+1, *y*−1/2, −*z*+1; (ii) −*x*, *y*−1/2, −*z*+1; (iii) *x*, *y*−1, *z*; (iv) *x*, *y*+1, *z*.
